# The Usefulness of Fine Needle Aspiration Cytology in the Management of Parotid Gland Masses at a Tertiary Academic Hospital

**DOI:** 10.1007/s12070-023-03685-6

**Published:** 2023-06-15

**Authors:** Fanelesibonge B Mdletshe, Thifhelimbilu E Luvhengo, Dipuo Masege

**Affiliations:** 1 Department of Otorhinolaryngology, School of Clinical Medicine, Faculty of Health Sciences, University of the Witwatersrand, 7 York Road, Parktown, Johannesburg, 2193 Republic of South Africa; 2 Department of Surgery, School of Clinical Medicine, Faculty of Health Sciences, University of the Witwatersrand, 7 York Road, Parktown, Johannesburg, 2193 Republic of South Africa; 3 Division of Otorhinolaryngology Head and Neck Surgery, Department of Neurosciences, 1st Floor, Office 7, Friends of Baragwanath Building, , Chris Hani Baragwanath Academic Hospital and University of the Witwatersrand, PO Bertsham, Johannesburg, 2013 Republic of South Africa

**Keywords:** Concordance, Fine Needle Aspiration Cytology, Milan Criteria, Parotid, Tumours

## Abstract

**Background:**

Fine needle aspiration cytology (FNAC) is an integral part of the preoperative work-up of parotid tumours.

**Aim:**

To determine the rate of concordance between FNAC and histology following parotidectomy.

**Methods:**

A review of records of patients who had parotidectomy which was preceded FNAC was done. Data collected included patients’ demography, presenting symptoms and clinical signs; cytology and post-operative histology results.

**Results:**

Seventy-seven records were found and 14 were excluded. Forty-five (71%: 45/63) of the tumours were benign, 21% (13/63) malignant and 8% (5/63) inflammatory lesions. Forty-one (91.1%: 41/45) of the benign tumours had concordance between FNAC and final histology. Seven (63.6%: 7/11) of FNAC diagnosed malignancies were confirmed on histology.

**Conclusion:**

Around 71% of parotid masses were benign. Painful masses are more likely to be malignant and FNAC is more reliable for the diagnosis of pleomorphic adenoma than rare benign and malignant tumours of the parotid gland.

## Introduction

Neoplasms of the salivary gland are rare and constitute around 3–6% of all head and neck tumours, and majority of them occur in the parotid gland [1]. Over 80% of neoplasms of the parotid gland are benign [2]. Pleomorphic adenoma and Warthin’s tumour are the most common benign tumours of the parotid gland [2, 3]. The common malignant tumours of the parotid gland are mucoepidermoid carcinoma, adenoid cystic carcinoma and acinic cell carcinoma [1, 2]. Rare malignant tumours of the parotid gland include adenocarcinoma, squamous cell carcinoma, lymphoma [4] and carcinoma ex-pleomorphic adenoma [5].

There is a similarity between the clinical presentation of benign and malignant lesions of the parotid gland [1, 6]. Facial nerve involvement, a painful mass, cervical lymphadenopathy in a patient who has a parotid tumour is/are highly suggestive of malignancy. Facial nerve palsy and enlargement of cervical lymph nodes may however occur in patients who had benign diseases of the parotid gland such as pleomorphic adenoma, odontoma and granulomatous parotitis [7–9]. Fine needle aspiration cytology (FNAC) is key in the preoperative work-up of parotid masses [6, 10, 11]. The result of FNAC is used to guide management including the need for additional investigations [12, 13].

The sensitivity and specificity of FNAC in benign and malignant tumours of the parotid gland is 71-100% and 33 − 100%, respectively [10, 12, 14, 15]. The positive and negative predictive value, and accuracy of FNAC for parotid masses ranges from as low as 33% to over 80% [14, 15]. Although FNAC has a high negative predictive value, it is less helpful for pre-operative subclassification of primary or metastatic malignancies of the parotid gland [14]. The diagnostic yield of FNAC is significantly low in Warthin’s tumour, low-grade mucoepidermoid carcinoma and rare malignant tumours of the parotid gland [16–22]. An incorrect FNAC result may lead to a delay in surgical intervention or performance of an unnecessary parotidectomy such as for tuberculous parotitis [23].

The difficulties associated with the interpretation and reporting of results together with the heterogeneity of salivary gland tumours have necessitated the development of systems to standardize reporting results following FNAC of salivary gland tumours [24]. The Milan System for reporting salivary gland cytopathology is the most commonly used system globally [14, 16, 24]. The Milan System categorizes FNAC result into non-diagnostic (I), non-neoplastic (II), atypia of unknown undetermined significance (III), benign neoplasm (IVa), neoplasm of uncertain malignant potential (IVb), suspicious of malignancy (V) and malignant (VI).

In our setting FNAC is done free-hand and the Milan System has not yet been adopted. The researchers have dealt with patients who had radical parotidectomy based on pre-operative FNAC result when surgery was not actually necessary. The aim of the study was to determine the rate of concordance between pre-operative FNAC and histology following parotidectomy.

## Materials and methods

Records of patients 18 years and older who had a preoperative FNAC followed by parotidectomy for parotid gland mass over a 5-year were reviewed. Patients who had redo-parotidectomies were excluded. Categorical data such as gender, diagnosis and type of surgical procedure were summarised using counts and percentage. The average or median was used to aggregate continuous data such as age. Sensitivity, specificity, accuracy, positive predictive value and negative predictive were calculated. The chi-square test was used for comparison of categorical data. Statistical significance was set at a p-value below 0.05.

## Results

A total of 76 records of patients who had parotidectomy were accessed of which 13 were excluded. Four (30.8%: 4/13) of the records were excluded as the patients were below the age of 18 years, 2/13 (15.4%) had re-do parotidectomy, 4/13 (30.8%) did not have histology results and in 3/13 (23.19%) FNAC results could not be traced. The final sample was made up of 63 patients that had parotidectomy which was preceded by FNAC. Thirty-three (52%: 33/63) of the patients were males and their mean age was 43.3 ± 12.9 years (range: 23–74 years). Forty-five (71.4%: 45/63) of the patients were below the age of 51 years (Fig. 1).

Eight (12.7%:8/63) patients presented with a painful mass and 1.6% (1/63) had evidence of paralysis of the facial nerve. Forty-eight 76.2% (48/63) of the masses had a solid architecture (Table 1). The most commonly reported category as per the Milan system was Category IVa (benign neoplasm) making up 47.6% (30/63) of the records and was followed by Category II at 31.7% (20/63) (Table 2). The diagnosis of isolated pleomorphic adenoma was captured in 27/63 (42.9%) of the records. Malignant tumours which were either confirmed or suspected on FNAC included adenoid cystic carcinoma, mucoepidermoid carcinoma and poorly differentiated carcinoma (Table 3).

**Table 1:** Clinical presentation and imaging findings in patients who had parotid masses (N = 63)

Parameter	Finding	Frequency
***Mass***	Yes	63 (100%)
***Nature of mass***	Complex	6 (9.5%)
	Cystic	9 (14.3%)
	Solid	48 (76.2%)
***Pain***	Present	8 (12.7%)
	Absent	55 (87.3%)
***Facial nerve palsy***	Yes	1 (1.6%)
	No	62 (98.4%)
***Other***	Previous stab injury to parotid gland	1 (1.6%)
	Recurrent sialadenitis and previous gunshot to parotid area	1 (1.6%)
	Skin ulceration	1 (1.6%)

**Table 2:** Breakdown of FNAC diagnoses according to Milan system (N = 63)

Category (Milan classification)	Number (Percentage)
Category I	6(9.5%)
Category II	20(31.7%)
Category III	0(0%)
Category IVa	30(47.6%)
Category IVb	0(0%)
Category V	3(4.8%)
Category VI	4(6.3%)
Total	63(100%)

**Table 3:** Breakdown of diagnostic findings following FNAC (N = 63)

Parameter	Frequency
Pleomorphic adenoma	27 (42.9%)
Lymphoepithelial cyst	16 (25.4%)
Pleomorphic adenoma or adenoid cystic carcinoma	2 (3.2%)
Adenoid cystic carcinoma	2 (3.2%)
Chronic sialadenitis	2 (3.2%)
Epidermal cyst	2 (3.2%)
Basal cell adenoma or pleomorphic adenoma	1 (1.6%)
Pleomorphic adenoma or mucoepidermoid carcinoma	1 (1.6%)
Warthin's tumour or pleomorphic adenoma	1 (1.6%)
Low grade mucoepidermoid carcinoma	1 (1.6%)
Poorly differentiated carcinoma	1 (1.6%)
Lipoma	1 (1.6%)
Inconclusive	6 (9.5%)
**Total**	**63 (100%)**

Forty-two (66.7%: 42/63) of the masses were histologically confirmed to be neoplastic whereas 7.9% (5/63) were inflammatory. Twenty-six (61.9%: 26/42) of the neoplastic lesions were pleomorphic adenoma. Lymphoepithelial cysts were confirmed in 75.0% (12/16) of the cases which were suspected following preoperative FNAC. All seven malignant tumours of the parotid gland which were suspected on pre-operative FNAC were confirmed on histology and the overall false negative rate was 36.4% (4/11). Three (27.3%: 3/11) of the malignant tumours were mucoepidermoid carcinomas (Table 4).

**Table 4:** Histology results following parotidectomy in patients who had pre-operative FNAC (N = 63)

Histological diagnosis	Frequency
Pleomorphic adenoma	26 (41.3%)
Lymphoepithelial cyst	8 (12.7%)
Mucoepidermoid carcinoma	3 (4.8%)
Basal cell adenoma	3 (4.8%)
Lymphoepithelial cyst and lymphoid hyperplasia	2 (3.2%)
Lymphoepithelial cyst and chronic sialadenitis	2 (3.2%)
Adenocarcinoma	2 (3.2%)
Adenoid cystic carcinoma	2 (3.2%)
Carcinoma ex-pleomorphic adenoma	2 (3.2%)
Chronic sialadenitis	2 (3.2%)
Epidermal inclusion cyst	2 (3.2%)
Acinic cell carcinoma	1 (1.6%)
Non-Hodgkin lymphoma	1 (1.6%)
Warthin’s tumour	1 (1.6%)
Dermoid cyst	1 (1.6%)
Lipoma	1 (1.6%)
Oncocytoma	1 (1.6%)
Chronic sialadenitis and reactive lymphoid hyperplasia	1 (1.6%)
Tuberculosis	1 (1.6%)
Necrotizing granulomatous inflammation, Ziehl-Neelsen stain negative	1 (1.6%)
**Total**	**63 (100%)**

Forty-one (91.1%: 41/45) the non-malignant lesions were correctly diagnosed as benign following preoperative FNAC. Seven (63.6%: 7/11) malignant tumours were correctly diagnosed pre-operatively following FNAC. One (5%: 1/20) malignant tumour was diagnosed in the 19–35 years age group compared to 20% (5/25) and 38.9% (7/18) each for the 36–50 years and the above 50 years age groups, respectively. The difference in the rate of occurrence of malignancies in the patients in the age groups was statistically significant (p-value = 0.032). None of the patients who had benign neoplasms complained of pain as compared to 38.5% (5/13) in patients who had malignant tumours, and the difference was statistically significant (p-value of 0.000) (Table 5). The overall positive predictive value was 100%( all seven of the cases assessed as malignant on FNAC were confirmed on histology; there were no false positive cases) and negative predictive value was 91%( 41/45 cases that were identified as benign on FNAC were confirmed as benign following parotidectomy; however, 4/11 cases that were missed on FNAC were confirmed malignant on histology).

**Table 5:** Relationship between histological findings and clinical findings

Symptom		Benign	Inflammatory	Malignant	p-value
**Pain**	Yes	0 (0.0%)	3 (60.0%)	38.5%	0.000
	No	45 (100.0%)	2 (40.0%)	61.5%	
**Facial palsy** **nerve**	Yes	0 (0.0%)	0 (0.0%)	7.7%	0.142
	No	100.0%	5 (100.0%)	92.3%	

## Discussion

The aim of the study was to ascertain the usefulness of fine-needle aspiration cytology (FNAC) in clinical decision-making for parotid gland masses. The study compared preoperative FNAC results with the post-operative histological findings. Among the key findings is that FNAC is highly reliable for diagnosing benign parotid gland tumours with specificity above 90% as compared to low specificity of 30% for malignant lesions.

Pleomorphic adenoma, Warthin’s tumour and lymphoepithelial cyst were as expected among the most commonly diagnosed causes of parotid masses [2, 25]. The most common malignant tumour of the parotid gland was mucoepidermoid carcinoma which is in keeping with what is reported in the literature [2, 25]. The higher rate of discordance between pre-operative FNAC and histology involved acinic cell carcinoma and other rare benign and malignant tumours of the parotid gland, which is not surprising especially in cases of low-grade tumours [21].

The study only consisted of adult patients whose mean age at presentation was 43.3 years. Although neoplasms of the parotid gland can occur in children, they are exceedingly rare [23] There was a significant relationship between age and final histology results and the incidence of malignant disease increased with age and this is in keeping with current literature [2]. All patients who were studied presented with a parotid mass. Only in one patient who had a malignant tumour had evidence of facial nerve paralysis. Facial nerve palsy is however sometimes seen in patients who have benign tumours such as pleomorphic adenoma [26]. Unlike the involvement of the facial nerve, a painful tumour is likely to be associated with an underlying malignancy as it was shown in the current study; which differs with the findings by Mallon and colleagues [14].

The most common neoplasm of the parotid in the current study was pleomorphic adenoma, which is like what is reported in the literature [2, 14, 27]. The next most common cause of parotid masses was lymphoepithelial cyst. High prevalence of lymphoepithelial cyst is likely to be due high incidence of HIV in the study population [28, 29]. The finding however underestimates the prevalence of lymphoepithelial lesions of the parotid gland as parotidectomy is not among the recommended treatment options for a lymphoepithelial cyst [30].

As expected, 21% of the neoplastic masses of the parotid gland were malignant tumours [27]. The most common malignant tumour was mucoepidermoid carcinoma which is in keeping with prior findings [2, 3]. Majority of malignancies were suspected on preoperative FNAC(7/11; 63.6%). None of acinic cell carcinoma, carcinoma ex-pleomorphic adenoma and lymphoma was diagnosed on FNAC. It is however known that FNAC may miss low grade mucoepidermoid carcinoma and the rare tumours of the salivary gland [18, 21, 22].

Granulomatous diseases accounted for 3,2% of the causes of parotid masses. Among the granulomatous diseases of the parotid gland is tuberculosis (TB) which was unfortunately only diagnosed following parotidectomy in the current study. Tuberculosis of the parotid gland is an uncommon condition even in countries where the disease is endemic [31]. Tuberculous parotitis may present as a hard mass mimicking malignancy [23]. A low index of suspicion for TB, along with an inconclusive FNAC result led to an unnecessary parotidectomy instead of medical therapy which is the recommended treatment [31]. It is however possible to diagnose TB of the parotid gland on FNAC as was demonstrated by Gupta and colleagues [32].

Other rare benign conditions such as lipomas were found on 1.6% patient in the study which is in keeping with the reported prevalence rate of 0.6–4.4% [33]. Lymphoma of the salivary glands is rare and account for 2–5% of all salivary gland tumours. Around 70–80% of lymphoma of the parotid gland occur in the parotid gland [34]. In our study, one case of non-Hodgkin’s lymphoma needed parotidectomy as FNAC result was inconclusive. Other authors have also reported a low probability of diagnosing basal cell adenoma [35], oncocytoma [8], low grade mucoepidermoid carcinoma [36], carcinoma ex pleomorphic adenoma [5], lymphoma [34], adenocarcinoma, acinic cell carcinoma [1, 18]. However, contrary to the reports FNAC was able to diagnose lipoma of the parotid gland [20].

Majority of malignant tumours in the current study were diagnosed following FNAC. The relatively high overall false negative rate of 36.4% might have been because salivary gland tumours in our setting are not managed by a dedicated multidisciplinary team [37]. Furthermore, FNAC in our setting is done free hand and not under ultrasound guidance. Our false negative rate of diagnosis of malignant tumours was however lower that the figures of 43.8% for mucoepidermoid carcinoma reported by a team led by Reerds [37].

## Limitations of the Study

The number of cases included in the study is relatively small. Salivary gland tumours are rare, and the size of samples included in majority of previous studies rarely have more than 100 patients. The study relied mainly on records over a period of five years and the challenge encountered was that some details were incorrectly entered, making it very difficult to trace both FNAC and histology results of each patient. This meant that with every incomplete or incorrect record, that patient could not be included in the study. Parotid gland masses are also managed by other departments such as general surgery, whose records were not reviewed. This further skew the sample it is not a true representation of all patients presenting with parotid gland masses to this academic centre. Only patients from one academic hospital were studied which introduces some bias into the study.

## Conclusion

Majority of parotid swellings occur in patients below the age of 51 years. Around 71% of parotid masses are due to benign neoplasms. A painful parotid tumour is more likely to be malignant. Although fine needle aspiration cytology is more reliable for the diagnosis of pleomorphic adenoma, it is less useful in the diagnostic work-up of malignant tumours of the parotid gland.

## Data Availability

Data will be made available when requested.

## Declarations

### Declarations

The study was self-funded and was done as part of mandatory requirement for Master of Medicine (Otorhinolaryngology) for the first author.
